# No Adverse Effect of Genetically Modified Antifungal Wheat on Decomposition Dynamics and the Soil Fauna Community – A Field Study

**DOI:** 10.1371/journal.pone.0025014

**Published:** 2011-10-17

**Authors:** Caroline Duc, Wolfgang Nentwig, Andreas Lindfeld

**Affiliations:** Institute of Ecology and Evolution, University of Bern, Bern, Switzerland; Argonne National Laboratory, United States of America

## Abstract

The cultivation of genetically modified (GM) plants has raised several environmental concerns. One of these concerns regards non-target soil fauna organisms, which play an important role in the decomposition of organic matter and hence are largely exposed to GM plant residues. Soil fauna may be directly affected by transgene products or indirectly by pleiotropic effects such as a modified plant metabolism. Thus, ecosystem services and functioning might be affected negatively. In a litterbag experiment in the field we analysed the decomposition process and the soil fauna community involved. Therefore, we used four experimental GM wheat varieties, two with a race-specific antifungal resistance against powdery mildew (Pm3b) and two with an unspecific antifungal resistance based on the expression of chitinase and glucanase. We compared them with two non-GM isolines and six conventional cereal varieties. To elucidate the mechanisms that cause differences in plant decomposition, structural plant components (i.e. C∶N ratio, lignin, cellulose, hemicellulose) were examined and soil properties, temperature and precipitation were monitored. The most frequent taxa extracted from decaying plant material were mites (Cryptostigmata, Gamasina and Uropodina), springtails (Isotomidae), annelids (Enchytraeidae) and Diptera (Cecidomyiidae larvae). Despite a single significant transgenic/month interaction for Cecidomyiidae larvae, which is probably random, we detected no impact of the GM wheat on the soil fauna community. However, soil fauna differences among conventional cereal varieties were more pronounced than between GM and non-GM wheat. While leaf residue decomposition in GM and non-GM wheat was similar, differences among conventional cereals were evident. Furthermore, sampling date and location were found to greatly influence soil fauna community and decomposition processes. The results give no indication of ecologically relevant adverse effects of antifungal GM wheat on the composition and the activity of the soil fauna community.

## Introduction

Since the first commercialised GM crops were released in 1994 [1], the area under cultivation of GM plants has been substantially amplified [2]. The global area cultivated with GM crops increased from 1.7 million ha in 1996 to 134 million ha in 2009 [3]. But the release of GM crops raises concerns about potential ecological and environmental risks. GM crops, planted in the field, will inevitably come into contact and interact with other plant species, microorganisms as well as animals that together perform several ecological processes such as biocontrol, pollination or decomposition [4]. It is of course not possible to assess all these aspects within one study and therefore we concentrated on decomposition and soil organisms. Beneficial soil fauna such as decomposers is a group of non-target organisms that should be considered as part of risk assessments due to their importance for the decomposition process of organic matter and due to their intense exposure to crop residues in the soil [5]. Soil fauna organisms could be affected by GM crops by feeding on living GM plant material (herbivores), on prey containing GM products (predators), on plant residuals (detritivores) or may get in contact with GM products via root exudates [6]. In earlier works about antifungal GM wheat, no detrimental effects were found on the performance of collembolans, dipteran larvae and enchytraeids or at least differences between GM and non-GM wheat were inconsistent and lay within differences among conventional wheat varieties [5], [7]–[9]. However, these studies were all done in the laboratory.

The present study assessed several questions and problems concerning GM wheat as wheat is one of the most important crops worldwide. Wheat fungal diseases greatly affect crop productivity, both in terms of quality and quantity [10], and require the economically and ecologically undesirable application of fungicides [11]. Powdery mildew is a ubiquitous disease and one of the main fungal pathogens in the field [12]. It is caused by the fungus *Blumeria graminis* f.sp. *tritici*, which affects the leaves of wheat and reduces wheat grain yield and flour quality [13].

We evaluated GM wheat plants with two different types of resistance. Two wheat varieties have a specific resistance gene against powdery mildew (Pm3b). These varieties express proteins, which detect pathogen-specific avirulence gene products (effectors) and generate a cascade of events leading to a resistance reaction that blocks further pathogen invasion [14], [15]. Those Pm3b antifungal wheat varieties were compared to their corresponding isolines, which were technically treated in the same way and are genetically identical to the GM varieties except for the presence of the transgene. Two other varieties were used with two unspecific active antifungal resistance mechanisms, which by expressing the hydrolytic enzymes chitinase and glucanase break down the fungal cell walls composed largely of chitin and glucan, thus limiting fungal growth [11], [12]. As comparison, the wild type variety from which the last mentioned GM wheat varieties derived was included. A further aim and difficulty in the risk assessment of GM crops is to detect changes outside the natural range of variability [16]. Therefore other conventional wheat varieties as well as varieties of the cereals barley and triticale were taken in consideration in this study. By doing that naturally occurring variance among conventional wheat varieties and other cereals can be evaluated and be used as a comparator to differences between GM and non-GM wheat.

This experiment focused on decomposition as a major function in sustainable agroecosystems and on the soil fauna as important decomposition regulators. Litterbag experiments are well suited since the decomposition process can be investigated as well as the soil fauna community involved. These functional and community aspects cannot be investigated in laboratory studies but require field experiments. However, there are only few works published about decomposition processes and the soil community structure in GM crop fields [17], [18] and none has been performed with GM wheat so far. Our field study, embedded into a unique three-year field experiment in Switzerland (www.konsortium-weizen.ch), therefore assesses several questions and problems concerning GM wheat for the first time.

We do not expect a direct impact of GM wheat varieties with the Pm3b resistance gene, since their specific resistance mechanism is triggered solely by the attack of powdery mildew. But GM wheat varieties, which express chitinase and glucanase, possibly have a direct detrimental impact on soil fauna and therefore on decomposition processes [19], since chitin and glucan are main structural components of the cell wall of fungi as well as of the exoskeleton of arthropods. Moreover, soil fauna and decomposition may be indirectly influenced by GM wheat. Genetic transformation may change plant metabolism and hence the composition of structural plant components, such as the lignin or cellulose content, as a result of randomly integrating a foreign gene into the plant genome [20]. Any transformation-related or pleiotropic change to the nutritional quality of crop residues could modify the composition and activity of soil organisms [21], altering in turn the decomposition rate [22]. We hypothesised that (1) soil fauna community does not differ between GM and non-GM wheat and hence (2) decomposition rate is not affected. Therefore we conducted two six-month litterbag studies in 2008/09 and 2009/10. Litterbags allow simultaneous collection of both community or taxonomic data (diversity and relative abundance of soil fauna) and information on ecosystem functions (decomposition) [23]. Beside soil fauna community and decomposition rate, C/N ratio and structural plant components (cellulose, hemicellulose and lignin) were considered. Furthermore, environmental factors, such as soil parameters and temperature were monitored, since they may have a strong impact on soil fauna community structure and decomposition rates [24].

## Results

### Soil fauna

In 2009 a total number of 40485 individuals belonging to 43 taxa were extracted from the litterbags (Table 1). More than 43% of all individuals extracted were mites, with Cryptostigmata (16%), Gamasina (14%) and Uropodina (9%) as dominant mite taxa. Springtails accounted for more than 27%, with Isotomidae (20%) as important collembolan family. Other taxa accounting for more than 5% were Enchytraeidae (Clitellata) and Cecidomyiidae larvae (Diptera) with 12% and 11%, respectively. These six dominant taxa appeared quite consistently over the six months of the experiment; only Cecidomyiidae larvae were considerably more frequent during the first two months and became less common during the last months. Other taxa were only transiently abundant such as the collembolan families Neelidae in December 2009 and Tullbergiidae in April 2010 and prostigmatid mites in April 2010.

**Table 1:** Total number of individuals of taxonomic units.

Individuals (no.)												
Taxa	Pm3b#1	Sb#1	Pm3b#2	Sb#2	A9	A13	Frisal	Toronit	Rubli	BWS26	Estana	Trado
Nematoda	0	2	2	3	3	3	3	5	3	1	2	4
Annelida												
Clitellata												
Lumbricidae	0	0	1	0	0	2	0	1	0	0	1	0
Enchytraeidae	417	329	410	336	434	614	462	441	466	378	462	458
Mollusca												
Gastropoda												
Arionidae	0	1	0	1	0	1	1	0	0	0	0	0
Arthropoda												
Chelicerata												
Arachnida												
Araneae												
Linyphiidae	3	0	1	0	0	2	2	0	3	2	0	1
Acarina												
Mesostigmata												
Gamasina	611	460	503	480	688	396	458	429	419	383	436	434
Uropodina	250	265	267	243	345	337	391	331	332	258	307	294
Prostigmata	125	118	151	114	105	123	185	186	164	95	132	118
Cryptostigmata	686	508	607	482	807	403	517	419	560	524	742	517
Crustacea												
Isopoda	1	0	1	0	2	0	3	1	1	3	0	0
Myriapoda												
Chilopoda	0	0	0	1	1	1	0	0	1	0	0	0
Diplopoda - Imagines												
Polydesmidae	0	0	0	1	0	2	0	0	1	1	2	1
Craspedosomatidae	0	0	0	1	0	2	0	0	1	1	2	1
Julidae	24	31	10	22	15	28	17	13	16	31	12	23
Diplopoda – Larvae												
Julidae	2	5	3	2	3	7	4	4	8	5	10	3
Pauropoda	2	6	10	4	12	8	2	2	15	0	6	7
Symphyla	0	0	0	0	1	1	0	0	1	0	0	1
Hexapoda												
Collembola												
Hypogasturidae	0	0	3	0	0	0	0	0	0	0	0	0
Tullbergiidae	59	84	96	82	58	60	52	90	49	51	134	60
Onychiuridae	6	0	9	4	13	13	19	1	34	24	144	78
Isotomidae	598	902	631	827	597	324	709	782	546	607	937	499
Entomobryidae	28	22	67	33	54	29	54	28	110	55	71	28
Neelidae	63	235	84	58	88	62	138	94	85	110	50	82
Sminthuridae	4	4	8	20	3	2	3	8	19	11	5	4
Insecta												
Psocoptera	1	1	0	0	1	1	2	1	1	1	2	2
Diptera – Larvae												
Chironomidae	102	50	191	42	64	38	15	20	75	64	101	93
Cecidomyiidae	221	406	352	556	405	409	475	372	691	321	246	332
Mycetophilidae	35	7	39	21	2	24	2	10	17	26	12	7
Scatopsidae	0	0	2	0	0	0	1	0	0	1	1	0
Dolichopodidae	4	1	2	20	11	0	1	5	3	2	2	5
Bibionidae	0	0	0	0	0	1	0	0	0	0	0	0
Empididae	2	0	1	0	1	1	1	2	0	3	2	1
Tipulidae	0	1	1	0	0	0	0	0	0	0	0	0
Rhagionidae	0	0	0	0	0	0	0	0	0	0	0	1
Stratiomyidae	0	1	0	1	1	0	1	0	1	0	0	0
Diptera – Imagines												
Stratiomyidae	0	0	0	0	0	0	0	0	1	0	0	0
Coleoptera – Larvae												
Cantharidae	0	1	0	0	0	0	0	0	0	0	0	0
Staphylinidae	10	20	10	10	8	4	4	17	15	11	8	12
Silphidae	0	0	0	0	0	0	0	1	0	1	0	0
Carabidae	0	3	4	3	2	1	0	2	3	3	2	2
Coleoptera – Imagines												
Staphylinidae	1	0	0	0	0	1	0	0	0	0	1	0
Phalacridae	0	0	0	0	1	0	0	0	0	0	0	4
Pselaphidae	0	0	1	0	0	0	0	0	1	0	0	1
Hymenoptera	0	0	0	0	0	0	0	0	1	0	0	0
Hemiptera												
Aphididae – Larvae	0	1	0	0	0	0	0	0	0	0	0	0
Thysanoptera	0	0	0	0	0	1	1	0	0	0	0	0
Total	3255	3464	3467	3367	3725	2899	3527	3265	3642	2972	3830	3072

Individuals were extracted from litterbags filled with leaves of different GM and non-GM wheat and other cereal varieties (2009 experiment).

**Figure 1:**
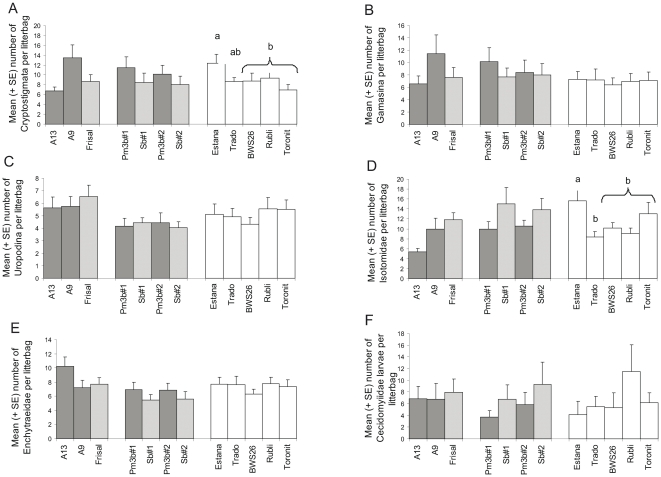
Mean (+ SE) number of individuals of the six most frequent taxa per litterbag (2009 experiment). GM group (*N* = 60 per variety): GM varieties are displayed dark grey and non-GM varieties bright grey. Conventional cereals (*N* = 60 per variety) are displayed white. Taxonomic groups are **A** Cryptostigmata, **B** Gamasina, **C** Uropodina, **D** Isotomidae, **E** Enchytraeidae and **F** Cecidomyiidae larvae. Different letters above the columns indicate significant differences (Tukey HSD test, *P*<0.05). Only significant differences are labeled.

**Figure 2:**
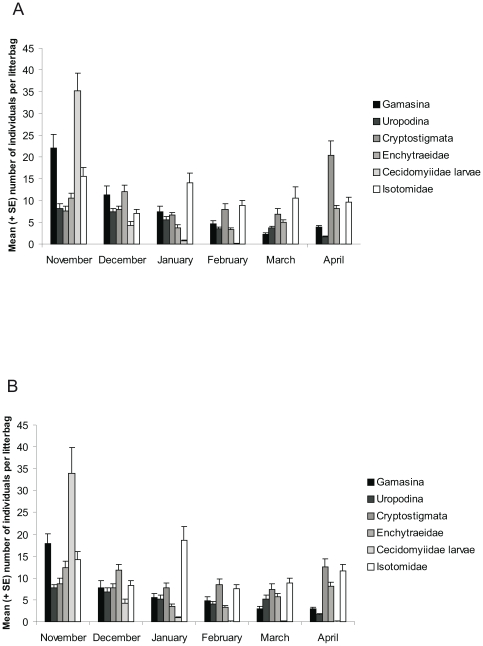
Mean (+ SE) number of soil organisms per litterbag for each month of the 2009 experiment. **A** GM group (*N* = 70 per sampling month). **B** Conventional cereals (*N* = 50 per sampling month). All six taxonomic groups are significantly different among the sampling months (A: LME, *P*<0.001, B: two-way ANOVA, *P*<0.05) with exception of Cryptostigmata, which did not vary among the months in the conventional crops.

In none of the months the presence of a transgene or the type of resistance affected the total amount of individuals extracted (N) or any of the taxonomic groups analysed in the GM group (*P*<0.05). For the complete data set, we also did not get any significant transgenic or resistance type effect for any of the above mentioned variables (Fig. 1, Table 2). However, all these variables varied significantly among the months (Fig. 2, Table 2). Furthermore, Cecidomyiidae larvae showed a significant transgenic/month interaction (Table 2). This suggests that the number of Cecidomyiidae larvae differed between GM and non-GM varieties, but differences were not consistent over months and no uniform pattern could be found. None of the other interactions were significant (Table 2).

**Table 2:** Complete data set analyses.

		Df 09	N	Cryptostigmata	Gamasina	Uropodina	Isotomidae	Enchytraeidae	Cecidomyiidae larvae	M 09	Df 08	M 08
GM group	Transgenic (Tr)											
	*F*	1,2	0.077	1.5778	0.038	0.121	2.344	0.808	2.416	0.818	1,2	0.248
	*P*		0.808	0.336	0.863	0.761	0.265	0.464	0.260	0.461		0.668
	Resistance type (Rt)											
	*F*	1,1	0.000	0.006	0.005	5.993	1.011	2.113	0.381	0.109	1,1	1.859
	*P*		0.997	0.953	0.956	0.247	0.498	0.384	0.648	0.797		0.403
	Month (Mo)											
	*F*	5,365	87.425	6.100	52.842	28.299	5.161	27.824	68.520	224.824	5,288	123.792
	*P*		<0.001	<0.001	<0.001	<0.001	0.001	<0.001	<0.001	<0.001		<0.001
	Tr × Rt											
	*F*	1,2	0.250	0.122	0.003	0.032	1.043	0.025	0.488	1.195	1,2	0.268
	*P*		0.667	0.761	0.960	0.875	0.415	0.889	0.557	0.388		0.656
	Tr × Mo											
	*F*	5,365	0.617	0.201	0.787	0.571	1.252	0.884	3.121	1.427	5,288	0.828
	*P*		0.687	0.962	0.560	0.722	0.284	0.492	0.009	0.214		0.530
	Rt × Mo											
	*F*	5,365	0.105	1.178	0.674	1.056	0.630	1.984	0.368	1.127	5,288	1.456
	*P*		0.991	0.320	0.634	0.385	0.677	0.080	0.871	0.345		0.204
	Tr × Rt × Mo											
	*F*	5,365	0.351	0.771	1.087	1.543	0.982	0.401	0.633	0.282	5,288	1.242
	*P*		0.882	0.571	0.367	0.176	0.429	0.848	0.674	0.923		0.290
Conventional cereal group	Species (Sp)											
	*F*	2,282	2.589	3.580	0.109	0.174	6.865	0.070	1.050	33.753	2,222	6.788
	*P*		0.077	0.029	0.897	0.840	0.001	0.932	0.351	<0.001		0.001
	Month (Mo)											
	*F*	5,282	42.671	1.197	15.519	13.753	2.585	20.530	29.185	182.517	5,222	72.025
	*P*		<0.001	0.311	<0.001	<0.001	0.026	<0.001	<0.001	<0.001		<0.001
	Sp × Mo											
	*F*	10,282	0.969	1.201	0.722	1.323	0.852	1.053	0.844	3.175	10,222	2.482
	*P*		0.470	0.290	0.704	0.218	0.579	0.577	0.587	0.001		0.008
Wheat group	Variety (Va)											
	*F*	2,162	0.988	1.077	0.009	0.716	0.179	0.737	2.289	0.294	2,126	5.098
	P		0.375	0.343	0.991	0.490	0.836	0.480	0.105	0.746		0.007
	Month (Mo)											
	*F*	5,162	29.087	2.843	7.401	6.311	0.950	17.286	57.364	74.074	5,126	29.921
	P		<0.001	0.017	<0.001	<0.001	0.451	<0.001	<0.001	<0.001		<0.001
	Va × Mo											
	*F*	10,162	0.757	0.313	0.352	0.686	1.288	0.855	1.366	1.008	10,126	0.767
	P		0.670	0.977	0.965	0.737	0.241	0.577	0.200	0.439		0.660

Results displayed for the total abundance of individuals (N), the six most abundant taxonomic groups and the decomposition rate (M 09) of the 2009 experiment and the decomposition rate (M 08) of the 2008 experiment for the GM group (LME, with transgenic (Tr), resistance type (Rt), month (Mo) and their interactions as factors), the conventional cereal group and the wheat group (two-way ANOVA, with species (Sp) or variety (Va), month (Mo) and their interaction as factors). Df indicates degree of freedom.

Some analysed parameters differed among the cereal species (barley, triticale and wheat) and among the wheat varieties (Bobwhite, Toronit and Rubli) in the month-by-month analyses. We found two significant species effects and a single variety effect. The total amount of individuals extracted (N) varied among the cereal species in February 2010 (*F*_2,282_ = 4.779, *P* = 0.013). Individuals were more abundant in barley and wheat than in triticale. In November 2009, the collembolan family Isotomidae was more abundant in barley than in wheat and triticale (*F*_2,282_ = 4.778, *P* = 0.013). In December 2010, the wheat group analyses revealed significantly less Cecidomyiidae larvae in Bobwhite than in Toronit (*F*_2,162_ = 3.953, *P* = 0.031). The complete data set analyses showed two significant species effects but no variety effect (Fig. 1, Table 2). Cryptostigmata showed (Fig. 1.A, Table 2) a higher abundance in barley than in wheat, but no difference between wheat and triticale or between triticale and barley. Isotomidae abundance varied among the cereal species (Fig. 1.D, Table 2) with more Isotomidae in barley than in wheat and triticale. The total amount of individuals found (N) tended also to differ among the cereal species (Table 2). There were marginally more individuals in barley than in the other cereal species. As in the GM group, the overall abundance of individuals (N) and the abundance of the analysed taxa varied among the months, with few exceptions (Fig. 2, Table 2). Cryptostigmata never showed any month effect in conventional cereals (Table 2). Isotomidae did not vary among months when we took only the wheat group data into consideration (Table 2). No interactions could be found for all studied parameters (Table 2).

### Decomposition

#### 2008

**Figure 3:**
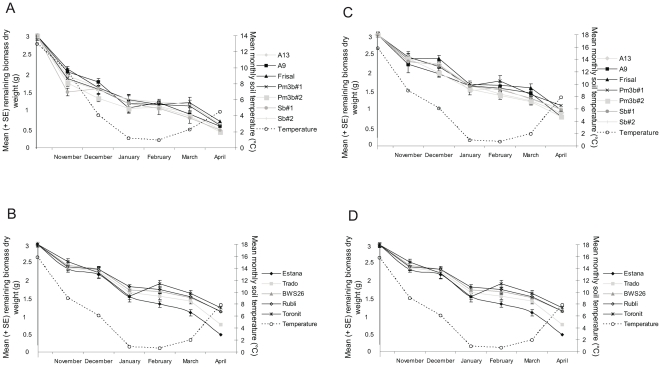
Mean (+ SE) remaining biomass dry weight for the different varieties from November 2008 to April 2009 (*N* = 8 per variety and month (A and B)) and from November 2009 to April 2010 (*N* = 10 per variety and month (C and D)). The dashed lines show the average monthly soil temperature curve at 5 cm depth. **A** and **C**: GM group. **B** and **D**: Conventional cereals. Asterisks show significant differences among the varieties (Tukey HSD test, *P*<0.05). Only significant differences are labelled.

Litter residues decreased significantly over time for the GM group as well as the conventional cereals (Fig. 3.A and B, Table 2, Table S2.A). Neither for the month-by-month analyses (Fig. 3.A) nor for the complete data analysis (Table 2) were differences found in decomposition rate (M) between GM and non-GM wheat or the two types of resistance.

The decomposition rate (M) differed more among the conventional cereal species and the different wheat varieties than between GM wheat and non-GM wheat. The monthly analyses revealed differences among the cereals for the first and the last two months of the experiment (Fig. 3.B). In November 2008 (*F*_2,37_ = 4.179, *P* = 0.023) wheat showed a faster decomposition rate than barley and triticale. However, in March (*F*_2,37_ = 5.098, *P* = 0.011) and April 2009 (*F*_2,37_ = 5.945, *P* = 0.006) barley decomposed faster than triticale and wheat (Table S1.A). Furthermore, the complete data set analysis revealed the same differences in decomposition among the conventional cereal species as in March and April 2009 with barley decomposing generally faster than triticale and wheat (Table 2). A significant species/month interaction (Table 2) was also found in the complete data set analysis. The wheat group analyses showed no differences in decomposition among the three varieties for each month separately (Fig. 3.B). However, in the complete data set analysis, the three wheat varieties differed in their decomposition rate (M) (Table 2), with Bobwhite decomposing slower than Rubli and Toronit.

The regression analyses revealed a single significant correlation between the chemical parameters and the decomposition rate (M). The C/N ratio is negatively correlated to decomposition: the higher the C/N ratio the slower the decomposition (*F*_1,574_ = 8.093, *P* = 0.005) (Figure S1, Table S4.A). The cellulose, hemicellulose, lignin and C/N ratio values are shown in Table 3.

**Table 3:** Structural component analysis.

Variety	Cellulose (% DW)	Hemicellulose (% DW)	Lignin (% DW)	C/N ratio
A13-Frisal	34.09	31.28	5.82	35.39
A9-Frisal	34.34	30.13	6.55	34.50
Frisal	32.41	33.41	7.39	29.73
Pm3b#1	33.87	29.95	6.81	35.01
Sb#1	34.00	30.31	7.71	26.86
Pm3b#2	32.04	31.63	8.06	27.31
Sb#2	33.47	30.35	7.09	30.81
Estana	37.24	31.34	5.35	33.08
Trado	36.35	31.18	5.68	37.42
Bobwhite	33.63	30.76	6.94	34.74
Toronit	34.94	29.60	6.84	31.06
Rubli	32.67	31.21	7.73	29.30

Cellulose, hemicellulose, lignin content (% dry weight) and CN ratio of the twelve varieties from the 2008 harvest.

#### 2009

Litter residues decreased significantly over time for the GM group as well as the conventional cereals (Fig. 3.C and D, Table 2, (Table S2.B). Neither for the month-by-month analyses (Fig. 3.C,) nor for the complete data analysis (Table 2) were differences found in decomposition rate (M) between GM and non-GM wheat or the two types of resistance.

The monthly analyses revealed differences among the cereals for the last three months of the experiment (Fig. 3.D). In February 2010, only wheat and barley differed from each other in their decomposition with barley decomposing faster than wheat (*F*_2,47_ = 8.609, *P*<0.001). In March 2010, triticale and wheat decomposed slower than barley (*F*_2,47_ = 11.860, *P*<0.001). In April 2010, barley decomposed the fastest and wheat the slowest (*F*_2,47_ = 28.600, *P*<0.001) (Table S1.B). Furthermore, the complete data set analysis revealed differences in decomposition among the conventional cereal species (barley>triticale>wheat) and a significant species/month interaction (Table 2). The wheat group analyses showed no differences in decomposition among the three varieties neither for each month separately (Fig. 3.D) nor for the complete data set (Table 2).

The regression analyses revealed no significant correlation between the chemical parameters and the decomposition rate (M). Nevertheless, lignin content tended to be correlated to decomposition (*F*_1,718_ = 2.753, *P* = 0.098) (Figure S2 Table S4.B). The higher the lignin content the slower the decomposition.

### Location effect

#### Soil fauna

Analyses revealed differences in the overall abundance of individuals (N) and in the abundances of the different analysed taxa among the five study blocks (Figure S3, Table 4). Monthly analyses showed differences among the experimental blocks for almost all variables (Table 4). Only Uropodina (November 2009, January 2010 and March 2010) and Echytraeidae abundances (February 2010 and March 2010) did not differ among the blocks (Table 4). The distribution patterns of the different dependent variables over the blocks appeared quite consistent for the six months analysed. Generally, the total amount of individuals (N) was higher in blocks 1 and 2 than in blocks 3, 4 and 5. Cryptostigmata and Gamasina mites tended to be more abundant in block 1 and 2 than in the other blocks. More Uropodina tended to be found in blocks 3, 4 and 5 than in blocks 1 and 2. Prostigmata mites (April 2010) appeared more in blocks 1 and 2 than in blocks 3, 4 and 5. Isotomidae had the highest abundance in block 3. The collembolan families Neelidae (December 2009) and Tullbergiidae (April 2010) were found in larger amount in blocks 1 and 2 than in blocks 3, 4 and 5. Enchytraeidae abundance was the highest in block 5. Cecidomyiidae larvae had the lowest number of individuals in block 3. The all data analyses showed differences in the total amount of individuals (N) and the six most dominant taxonomic group abundances among the blocks (Table 4).

**Table 4:** Location effect analyses.

		N	Cryptostigmata	Gamasina	Uropodina	Prostigmata	Isotomidae	Neelidae	Tullbergiidae	Enchytraeidae	Cecidomyiidaelarvae	M
November	Block											
	*F*_4,115_	11.003	17.368	46.504	1.456	NA	8.281	NA	NA	3.247	5.439	3.427
	*P*	<0.001	<0.001	<0.001	0.220	NA	<0.001	NA	NA	0.015	<0.001	0.011
December	Block											
	*F*_4,115_	18.961	3.071	73.945	4.247	NA	6.936	11.504	NA	21.750	7.6424	13.463
	*P*	<0.001	0.019	<0.001	0.003	NA	<0.001	<0.001	NA	<0.001	<0.001	<0.001
January	Block											
	*F*_4,115_	4.281	5.504	57.746	1.417	NA	21.333	NA	NA	31.216	NA	6.876
	*P*	0.003	<0.001	<0.001	0.233	NA	<0.001	NA	NA	<0.001	NA	<0.001
February	Block											
	*F*_4,115_	13.093	10.623	100.160	3.705	NA	2.935	NA	NA	1.429	NA	8.901
	*P*	<0.001	<0.001	<0.001	0.007	NA	0.024	NA	NA	0.229	NA	<0.001
March	Block											
	*F*_4,115_	3.751	4.310	35.613	1.072	NA	3.851	NA	NA	1.804	NA	5.767
	*P*	0.007	0.003	<0.001	0.374	NA	0.006	NA	NA	0.133	NA	<0.001
April	Block											
	*F*_4,115_	26.829	64.133	4.469	NA	17.077	9.259	NA	11.085	5.970	NA	0.912
	*P*	<0.001	<0.001	0.002	NA	<0.001	<0.001	NA	<0.001	<0.001	NA	0.460
Overall	Block											
	*F*_4,715_	13.132	29.795	142.890	5.729	NA	33.140	NA	NA	22.777	3.032	7.586
	*P*	<0.001	<0.001	<0.001	<0.001	NA	<0.001	NA	NA	<0.001	0.017	<0.001

Results displayed for all studied variables for the six months separately and for the complete data set of the 2009 experiment (one-way ANOVA with block as explanatory variable). NA indicates not analysed.

### Decomposition

In 2008, the month-by-month analyses indicated significant differences among the blocks in December 2008 (*F*_3,92_ = 2.983, *P* = 0.035) and in January 2009 (*F*_3,92_ = 5.670, *P* = 0.001). In December 2008 block 4 decomposed slower than block 3 and in January 2009 block 4 decomposed slower than the three other blocks (Figure S4.A, Table S3, Table S5.A). However, the complete data analysis could not reveal any significant differences in the decomposition rate among the study blocks for the 2008 experiment (Table S3).

In 2009, the decomposition rate (M) differed among the blocks in 2009. The monthly analyses revealed a faster decomposition rate in block 2 than in block 3 in November 2009 (Table 4). In December 2009, blocks 1 and 2 decomposed significantly faster than the three others (Table 4). In January and February 2010 more complex patterns were found. Block 1 decomposed much faster than blocks 3 and 5 (Table 4). In March 2010, all decomposition rates started to merge; only block 4 was significantly slower than block 1 and 2 (Table 4). In April 2010, we could not find any block differences in decomposition anymore (Figure S4.B, Table 4, Table S5.B). Over the complete data set, blocks 1 and 2 decomposed significantly faster than the three other blocks (Table 4).

## Discussion

### Soil fauna

Our field litterbag experiment revealed that senescent leaf litter of wheat, which has been genetically modified for improved fungal resistance, had almost no significant impact on the soil fauna community during an exposure of six months. Cecidomyiidae larvae only showed a significant transgenic/month interaction with sometimes Cecidomyiidae abundance being slightly higher, sometimes slightly lower in GM than in non-GM wheat. Because of the transient nature of these responses, it is difficult to distinguish between the statistical and the ecological significance [4] of the interaction. However, the monthly analyses could not reveal any significant transgenic effect for this taxonomic group. Furthermore, we had a huge variation in our data, probably due to the random distribution and limited dispersal abilities of these midges. Therefore, we assume this interaction to be rather stochastically than because of a transgene effect.

The total amount of individuals extracted (N) and some taxonomic groups differed occasionally among the different conventional cereals and among the different wheat varieties. It appears that soil fauna varied more within the conventional cereal group and the wheat group than within the GM group, meaning there are more differences among conventional wheat varieties and among different cereal species than between GM and non-GM wheat. Those findings accord with the results of previous works done on Bt corn where largely the same soil fauna community was found in their experiments as we did [25]–[28]. Mites and springtails are known to be dominant and very diverse groups in the soil [29] Enchytraeidae are also abundant in crops [30]. Though, we caught a large amount of Diptera larvae, mostly Cecidomyiidae, this was not the case in the studies from Zwahlen et al. [27] and Hönemann et al. [28]. However, one third of the Cecidomyiidae larvae are known to live as detritophages in the soil [31]. The few studies made on antifungal wheat draw the same conclusions as we do. Peter et al. [7] found that, in most cases, the number of *Drosophila melanogaster* progeny varied less between GM and non-GM Bobwhite varieties than between different conventional wheat varieties and Lindfeld et al. [8] did not find higher differences in the number of offspring of *Enchytraeus albidus* between GM and non-GM wheat than among conventional varieties. Likewise, Romeis et al. [5] did not find any transgenic effect on the population development of *Folsomia candida* (Collembola), but they detected a significant variety effect.

Generally, the six analysed taxonomic groups varied among the months for both the GM and the conventional cereal groups. Those seasonal dynamics appeared also in the studies from Zwahlen et al. [27] and Hönemann et al. [28]. The GM and the conventional cereal groups exhibited similar seasonal patterns. The distribution patterns of the different taxonomic groups may be shaped by the life cycle of the respective taxonomic group [32], by environmental and climatic conditions and by trophic interactions. Cecidomyiidae larvae were accounting for more than 30% of the individuals extracted in November and for less than 1% in April. Litterbags may have provided optimal microhabitats (moisture content and substrate accumulation) [23] for Cecidomyiidae larvae at the beginning of the experiment. By temperature drop in December, Cecidomyiidae larvae may have entered in diapause and may have overwintered as diapausing cocooned larvae. The end of overwintering diapause is regulated by temperature and some weeks at above 3°C are required to terminate diapause [33]. Cecidomyiidae larvae were probably still in diapause during our last sampling month (April), not active and hence not found in the litterbags. Enchytraeidae are dependent on climatic conditions. They increase rapidly when the conditions are favourable and are strongly reduced by frost [34]. In January and February, their abundance was low, probably because of the deep temperatures and the drought. Gamasina and Uropodina abundances decreased from November to April and followed similar dynamics as those found by Zwahlen et al. [27]. Soil biodiversity may potentially be affected by interactions within trophic levels (competition) or by direct trophic interactions such as predation [35]. Gamasina are generalist predators and therefore may have a strong impact on other taxa [36]. A decrease in Gamasina abundance could explain an increase of other taxonomic groups. Isotomidae were the most abundant group in January. Because Gamasina feed on Isotomidae [37] and were highly reduced in January, this may have resulted in an increase of this collembolan family. Nonetheless, Isotomidae became less abundant in the following months despite the low abundance of predatory mites. This might be explained by the insufficient moisture conditions [38], due to the rather scarce precipitation events. Cryptostigmata only barely varied among the sampling months and showed even no significant seasonal fluctuations in the conventional cereal group. This taxonomic group seems to be rather robust towards biotic and abiotic changes [39]. Moreover, soil humidity is only of minor importance for the density and community structure of Cryptostigmata [38]. In the GM group, Cryptostigmata mites increased in early spring, but not as much as in the study of Zwahlen et al. [27] who assumed that Cryptostigmata abundance may be controlled by Gamasina. However, a study from Peschel et al. [37] revealed that Cryptostigmata are protected by their thick cuticle and accordingly live in a relatively enemy-free environment. Only juveniles which are still weakly sclerotisised can be eaten [40].

### Decomposition

In both years conventional cereals differed in decomposition patterns, while GM and non-GM wheat varieties were more similar. This indicates that the decomposition rate of the antifungal GM wheat varieties assessed in the present study lie well within the range of variation found among commonly used conventional cereals. We could not detect any peculiar decomposition patterns of GM wheat. No previous studies investigated the decomposition of GM antifungal wheat so comparisons are not possible. However, an experiment done on chitinase transgenic silver birch did also not indicate any transgenic effect on litter decomposability in the soil [41], but we have to be careful with those kinds of comparison since transformation effects may vary from one GM plant to another and even similar proteins may differ in their degradability [17].

Organic matter decomposition and nutrient cycling are regulated by resource quality, decomposer community and environmental conditions [42]. These parameters may have driven the differences in decomposition among the conventional cereals in 2008 and 2009. Except for November 2008, which caused the month/species interaction, there was a clear trend of barley being decomposed faster than wheat and triticale. Since GM wheat showed no different decomposition patterns from non-GM wheat other factors than transgene products seem responsible. The differences among conventional cereals in both years and the conventional wheat varieties (Bobwhite, Rubli and Toronit) in 2009 support this assumption. Structural plant components and C/N ratio greatly affect palatability for decomposers and hence decomposition processes [43], [44]. Easily degradable crop varieties form better food resources. Lignin is a major structural component of plant cells, which confers strength, rigidity and impermeability to water [45]. Lignin is a stable, hard to degrade compound, therefore lignin degradation is most likely the rate limiting step for decomposition [46]. Higher lignin content may have tended to reduce decomposition in the soil. However, none of the chemical components analysed were significantly linked to decomposition, but in 2009 lignin tended to be correlated to litter decomposition. Barley had the lowest mean lignin content and showed with exception in November 2008 the fastest decomposition, while wheat had the highest lignin content and decomposed at the slowest rate within the conventional cereal group.

Differences in crop chemical composition could also modify the dynamics of the structure and activity of soil organisms [21]. The total amount of individuals extracted (N) tended to be higher in barley than in the other cereal species. Generally, the more soil organisms the faster the decomposition [42]. Santos and Whitford [47] demonstrated that exclusion of soil fauna highly reduces decomposition. Soil fauna increases the accessibility of detritus to microbes, e.g., by fragmentation of litter, increasing substrate surface for bacteria and fungus, which perform the main part of the decomposition [48]. Furthermore, Cryptostigmata and Isotomidae abundances differed significantly among conventional cereals. Those taxonomic groups are not directly implied in the decomposition process, since they are not primary decomposers [49]. However, they may stimulate microbial activity by grazing on soil microbes [50]. Therefore, differences in the chemical composition of the plants and in the soil fauna community may together explain variation in decomposition rate (M) among the conventional cereals.

### Location effect

Except for December 2008 and January 2009 no block effect on decomposition rate was detectable in the first study year whereas in the second year blocks 1 and 2 on the one side and blocks 3, 4 and 5 on the other side differed in soil fauna community and decomposition. This can be explained by the experimental design in the two years. The four blocks in 2008 were arranged close together whereas in 2009 blocks 1 and 2 and blocks 3, 4 and 5 were arranged apart. Differences in 2009 for soil fauna and decomposition were more pronounced among the study blocks as between GM and non-GM wheat. Our field in 2009 provided some small-scale heterogeneity with respect to soil properties (Table S6). Soil conditions can differ over short distances, which affects the local distribution of the different taxa and hence decomposition processes [51]. Furthermore it has to be mentioned that the preceding crops differed between blocks 1 and 2 on the one hand and blocks 3, 4 and 5 on the other hand. The preceeding crops themselves and the attended agricultural practices might have a formative effect on the soil fauna community found in the blocks [52]. The preceding crops were grass on blocks 1 and 2 and maize on blocks 3, 4 and 5. Generally, more individuals were found in blocks 1 and 2 than in blocks 3, 4 and 5. Moreover, Gamasina and Cryptostigmata were found in greater abundances in the first two blocks than in the last three ones. Soil parameter analyses revealed that blocks 1 and 2 were more alkaline and had lower clay content than blocks 3, 4 and 5 (Table S6). Indeed, the higher the pH [40] and the lower the soil clay content the greater the soil fauna abundance and activity [25]. However, Uropodina, Isotomidae, Enchytraeidae and Cecidomyiidae larvae occurred broadly in higher proportion with a lower pH. Gamasina and Cryptostigmata may have constrained the other taxa to blocks 3, 4 and 5 by competition and predation interactions [29]. The distribution of the different taxa over the blocks is shaped by complex interactions among many parameters, such as biotic interactions, climate and soil properties making further explanations too difficult. Decomposition tended to be faster in blocks 1 and 2 than in blocks 3, 4 and 5. As already mentioned, Gamasina mites were more abundant in blocks 1 and 2 than in the three other blocks and are important predators in the soil [49]. Hedlund & Sjögren Öhrn [53] demonstrated that tritrophic interactions enhance decomposition. Thus, predators may increase decomposition rate indirectly since microorganisms were not overgrazed by soil fauna. Furthermore, soil properties may also drive decomposition. Sandy soils may provide large pore space and high oxygen content, offering good conditions for microbial growth and consequently increase decomposition rate [54]. Those positive soil conditions are met in blocks 1 and 2 (Table S6).

### Conclusion

This is the first study about the potential impact of antifungal GM wheat on the soil fauna community and decomposition dynamics in the field. Despite a single significant transgenic/month interaction, which is rather stochastical than because of a transgene effect, our results revealed that soil fauna and decomposition are not affected by GM wheat. However, we used different conventional cereals and wheat varieties and we detected that both soil fauna and decomposition differed significantly among the conventional varieties. This emphasises how important it is to include not only GM plant varieties and corresponding isolines into risk assessment studies and compare them but conventional varieties and in our case other cereals, too. Only this additional integration allows to estimate naturally occurring variance as baseline comparators and thus the relevance of differences between GM and non-GM plants for ecosystem functioning. Sampling date and location were found to greatly influence soil fauna community and decomposition processes. Hence, parameters, such as temperature, precipitation and soil properties should be taken into consideration when assessing the influence of GM plants on soil fauna and decomposition. Field conditions cannot be simulated in the laboratory and hence laboratory results potentially vary from field results or are even reverse [55]. Thus, field experiments are crucial additions to ERAs for the evaluation of environmental consequences.

## Methods

### Ethics Statement

All necessary permits were obtained for the described field studies. Permits were given by the Federal Office for the Environment FOEN (http://www.bafu.admin.ch/) to a group of scientists to which we belong, the wheat cluster (http://www.wheat-cluster.ch/), which is part of the National Research Programme 59 (http://www.nfp59.ch/).

### Study site

The field study was conducted from October 2008 to April 2009 and October 2009 to April 2010 on the experimental field of the Agroscope Reckenholz-Tänikon Research Station (ART), in Zurich-Reckenholz. In 2008 we had a total of 48 plots (1.08×7.0 m) arranged in four blocks and in 2009 a total of 60 plots (1.08×3.0 m) arranged in five blocks. One block contained twelve plots each with a different cereal variety. The plots were separated by a one meter wide strip sown with grass. Soil types and pH were analysed in a laboratory at ART. Soil samples in June 2008 and 2009 were taken in all four and all five blocks, respectively. Each pooled sample consisted of ten samples taken randomly distributed over the respective block. Sampling depth was 0–20 cm. Precipitation and soil temperature at 5 cm depth were measured daily during the experimental periods.

### Plants

For our field experiment we used four GM wheat varieties and eight non-GM cereal varieties. Two GM varieties (Pm3b#1 and Pm3b#2) derived from the conventional Mexican wheat variety Bobwhite SH 98 26 (in the following referred to as Bobwhite), with a race specific resistance against powdery mildew. Their two corresponding isolines (Sb#1 and Sb#2) were included in the experiment as control lines. We also analysed two other GM varieties (A9 and A13) containing the antifungal barley seed chitinase or chitinase and β-1,3-glucanase and the non-transformed variety Frisal as a control from which they are derived. Additionally, Toronit and Rubli as conventional commercialised Swiss wheat varieties, the above mentioned Bobwhite and two other commonly used cereal varieties: a spring triticale (Trado) and a spring barley variety (Estana) were used in this experiment. The Pm3b-varieties and their isolines were bred at the University of Zurich and the Frisal-derived GM varieties at the ETH-Zurich. The GM varieties are experimental varieties and not suitable for commercialisation. For detailed information about the GM Pm3b- and A-varieties used see Srichumpa et al., 2005, Yahiaoui et al., 2006, Zeller et al., 2010 [15], [16], [56] and Bliffeld et al., 1999, Bieri et al., 2003 [12], [13], respectively. The trial was sown on March 30^th^, 2008 and on March 19^th^, 2009, respectively. No insecticide was applied on our plots. Harvest started on August 4^th^, 2008 and on July 28^th^, 2009 and was finished 10 and 9 days later, respectively. All experimental material was harvested by hand and dried for 48 hours in a ventilated drying chamber at 34–38°C.

### Litterbags

Litterbags made of polyethylene mesh (15×15 cm, 7 mm mesh size) were filled with 3 g dry weight of senescent leaves, each of them with one cereal variety. In mid-October 2008 and 2009, twelve litterbags per plot, filled with the respectively harvested plant material, were buried in a horizontal position at a depth of 5 cm. A total amount of 576 litterbags ( = 8 repetitions × 12 varieties × 6 months) in 2008 and 720 litterbags ( = 10 repetitions × 12 varieties × 6 months) in 2009 were buried in the field. Two litterbags per plot were randomly chosen and collected every month from mid-November 2008 to mid-April 2009 and mid-November 2009 to mid-April 2010, resulting in eight and ten litterbags per variety and sampling date, respectively. When collected from the field, litterbags were placed separately in plastic bags to avoid loss of plant material. The litterbags were then used for identification and analysis of the soil invertebrate community and to assess the decomposition.

### Soil fauna

In both years the collected litterbags were put into a MacFadyen-Extractor [56] for three days to extract the soil fauna. Individuals were collected in vials filled with isopropanol. In 2009 animals were identified on family level with some exceptions, which were identified on a higher taxonomic level.

### Decomposition

After extraction of the soil fauna, the plant residues from the litterbags were washed with water to remove soil particles, roots and other non-plant residue material. A fine mesh sieve (2 mm) was used during the procedure to avoid loss of plant material. All samples were dried at 40°C for seven days and the remaining dry weight was recorded for both years. The decomposition rate was then calculated as the ratio of the remaining dry weight of the sample and the initial dry weight. The contents of hemicellulose, cellulose, lignin and the C/N ratio of all cereal varieties were taken from an earlier study by Lindfeld et al. [8].

### Statistical analyses

Analyses were conducted in R 2.3.1 [57]. The analyses were performed over the complete data set to check for general differences in the studied parameters among the varieties. Data for each month were then analysed separately. Before performing any statistical analysis, all dependent variables were checked for normal distribution using the Shapiro-Wilk-Test. If needed, the variables were log- or square root-transformed to meet model assumptions. For statistical reasons, we had to analyse the GM varieties and their corresponding wild type and isolines separately from the conventional varieties. The linear mixed effects model (LME), described below, needs a paired design, which is not given for the conventional wheat varieties, barley and triticale, respectively.

For the first part of our analyses we used seven of the twelve experimental varieties: Pm3b#1, Pm3b#2, Sb#1, Sb#2, A9, A13 and Frisal (GM group). The four Bobwhite-derived varieties were divided into two groups, Pm3b#1 with Sb#1 and Pm3b#2 with Sb#2. We made a third group including A9, A13 and Frisal. These seven varieties were then separated in transgenic (the Pm3b- and the A-varieties) and non-transgenic (Frisal and the Sb-isolines). The varieties were also distributed in two different resistance types: specific resistance (the Pm3b-varieties and their corresponding isolines) and unspecific resistance (the A-varieties and Frisal). To test for differences in soil fauna community and decomposition between antifungal wheat and non-GM wheat, we used a linear mixed effects model (LME). For analyses of the complete data set, month, resistance type, transgenic and their interactions were fixed factors and variety, group and location (block number) random factors. Data for each month were analysed using the same linear mixed effects model procedure as described above with the exception of the month variable which was not included in the analyses. We conducted the above-described analyses for the decomposition rate (M) in 2008 and for the total amount of individuals extracted (N), the decomposition rate (M) and the most abundant taxonomic groups in 2009. Only soil fauna groups that accounted for more than 5% of the total amount of individuals extracted were considered: Cryptostigmata, Gamasina, Uropodina, Prostigmata (Acarina), Isotomidae, Neelidae, Tullbergiidae (Collembola), Enchytraeidae (Clitellata) and Cecidomyiidae larvae (Diptera).

To test for differences among the five different conventional cereal varieties, we proceeded in two steps. First we checked for differences among the different cereal species: barley, triticale and wheat (conventional cereal group). Then we analysed separately the three wheat varieties, looking for differences among Toronit, Rubli and Bobwhite (wheat group). We used a one-way ANOVA, with species or variety as factor for each month and a two-way ANOVA, with species or variety, month and their interaction as fixed variables for the complete data set. Each ANOVA was followed by a Tukey HSD multiple comparison post hoc test. The same dependent variables as in the above mentioned LME were analysed. This second part of the statistical analyses was useful to get a better idea on the natural variation among the different conventional cereals and the different wheat varieties.

Furthermore, we checked for a potential relationship between the composition of structural plant components of the different cereal varieties and the decomposition rate (M) of 2008 and 2009. We used the mean hemicellulose, cellulose, lignin and C/N ratio values for each variety and we performed a regression analysis. Finally, all dependent variables were tested for a location effect. We performed a one-way ANOVA followed by a Tukey HSD test for all twelve varieties together for the complete data set and each month separately. Block number was used as explanatory variable.

## Supporting Information

Figure S1 **Correlation of decomposition rate M of the 2008 experiment and chemical parameters of all experimental varieties** **(*N* = 48 per variety).** **A** Lignin **B** Cellulose **C** Hemicellulose and **D** C/N ratio. DW indicates dry weight.
(TIFF)

Figure S2 **Correlation of decomposition rate M of the 2009 experiment and chemical parameters of all experimental varieties** **(*N* = 48 per variety).** **A** Lignin **B** Cellulose **C** Hemicellulose and **D** C/N ratio. DW indicates dry weight.
(TIFF)

Figure S3 **Block effect on soil fauna.** Mean (+ SE) number of individuals per litterbag in the different blocks of the 2009 experiment (*N* = 144 per block). Letters above the columns indicate significant differences in taxa abundance among the blocks (Tukey HSD test, *P*<0.05).
(TIFF)

Figure S4 **Block effect on decomposition.** Mean (+ SE) remaining biomass dry weight in the blocks from November 2008 to April 2009 (**A**) (*N* = 24 per block and month) and from November 2009 to April 2010 (**B**) (*N* = 24 per block and month) and average monthly soil temperature curve at 5 cm depth. Asterisks show significant differences among the blocks (Tukey HSD test, *P*<0.05). Only significant differences are labelled.
(TIFF)

Table S1 **Decomposition rate analysis.** Results of the decomposition rate (M) analyses for the six months separately for the GM group (LME, with transgenic (Tr), resistance type (Rt) and their interaction as factors), the conventional cereal group and the wheat group (one-way ANOVA, with species or variety as factor). **A** 2008 experiment. **B** 2009 experiment.
(DOC)

Table S2 **Mean (+ SE) remaining biomass dry weight of the different varieties.** Results displayed from November 2008 to April 2009 (*N* = 8 per variety and month) and from November 2009 to April 2010 (*N* = 10 per variety and month). A GM group. B Conventional cereals. Different letters show significant differences among the varieties (Tukey HSD test, *P*<0.05). Only significant differences are labelled.
(DOC)

Table S3 **Location analyses for the decomposition rate (M).** Results displayed for the six months separately and for the complete data set of the 2008 experiment (one-way ANOVA with block as explanatory variable).
(DOC)

Table S4 **Structural component analyses.** Regression analysis between cellulose, C/N ratio, hemicellulose or lignin and decomposition rate) for the twelve varieties together. A 2008 experiment. B 2009 experiment.
(DOC)

Table S5 **Mean (+ SE) remaining biomass dry weight of the different blocks.** Results displayed from November 2008 to April 2009 (*N* = 8 per variety and month) and from November 2009 to April 2010 (*N* = 10 per variety and month). A 2008 experiment. B 2009 experiment. Different letters show significant differences among the varieties (Tukey HSD test, *P*<0.05). Only significant differences are labelled.
(DOC)

Table S6 **Soil parameters analyses.** Done for the different blocks. **A** 2008 experiment. **B** 2009 experiment.
(DOC)
