# Supplementary figures and images for: No Adverse Effect of Genetically Modified Antifungal Wheat on Decomposition Dynamics and the Soil Fauna Community – A Field Study
Source: PLoS One. 2011 Oct 17;6(10):e25014. doi: 10.1371/journal.pone.0025014 (PMC3197184; doi:10.1371/journal.pone.0025014)

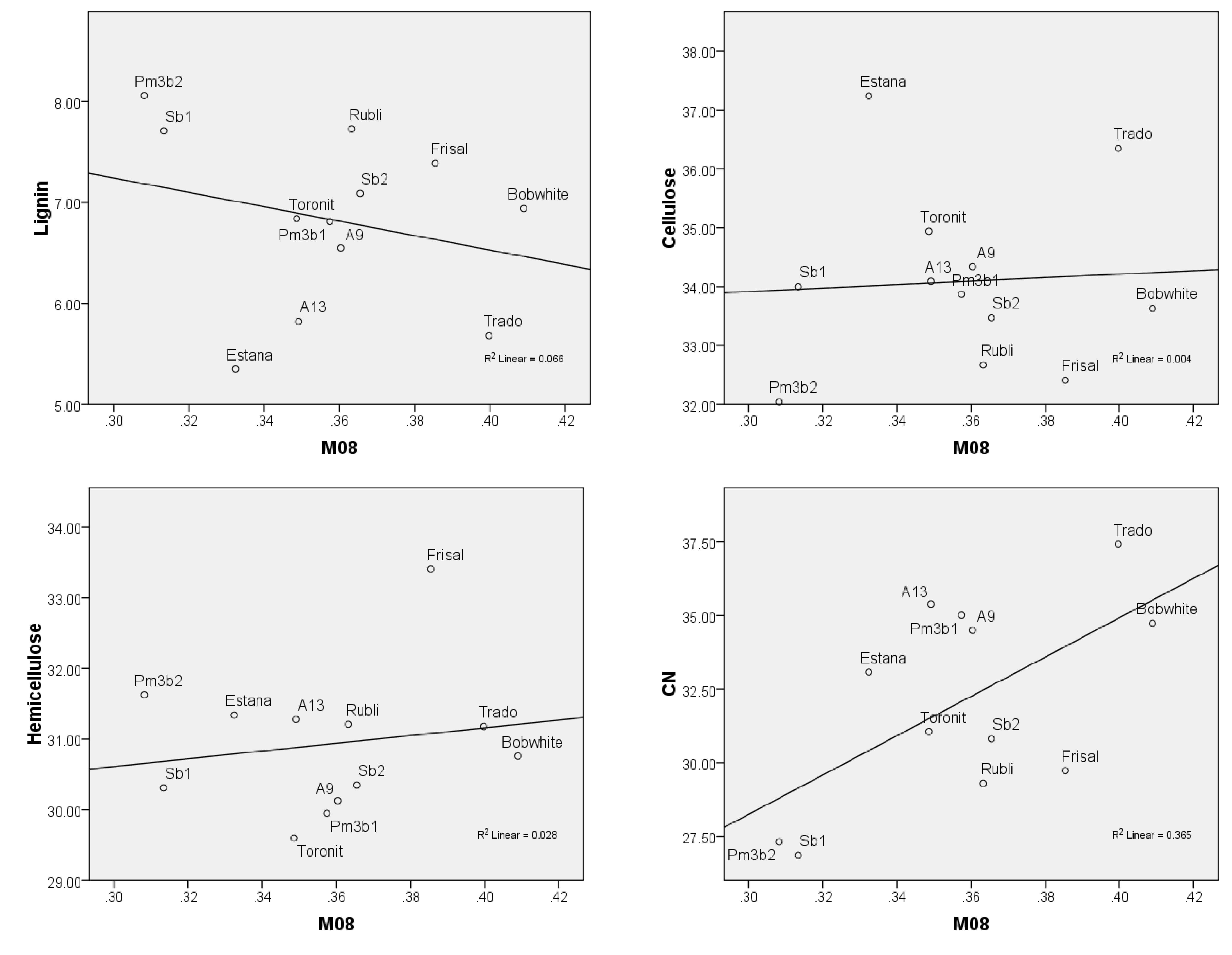

Supplement: Figure S1 — Correlation of decomposition rate M of the 2008 experiment and chemical parameters of all experimental varieties (N = 48 per variety). A Lignin B Cellulose C Hemicellulose and D C/N ratio. DW indicates dry weight. (TIFF) [file pone.0025014.s001.tiff]

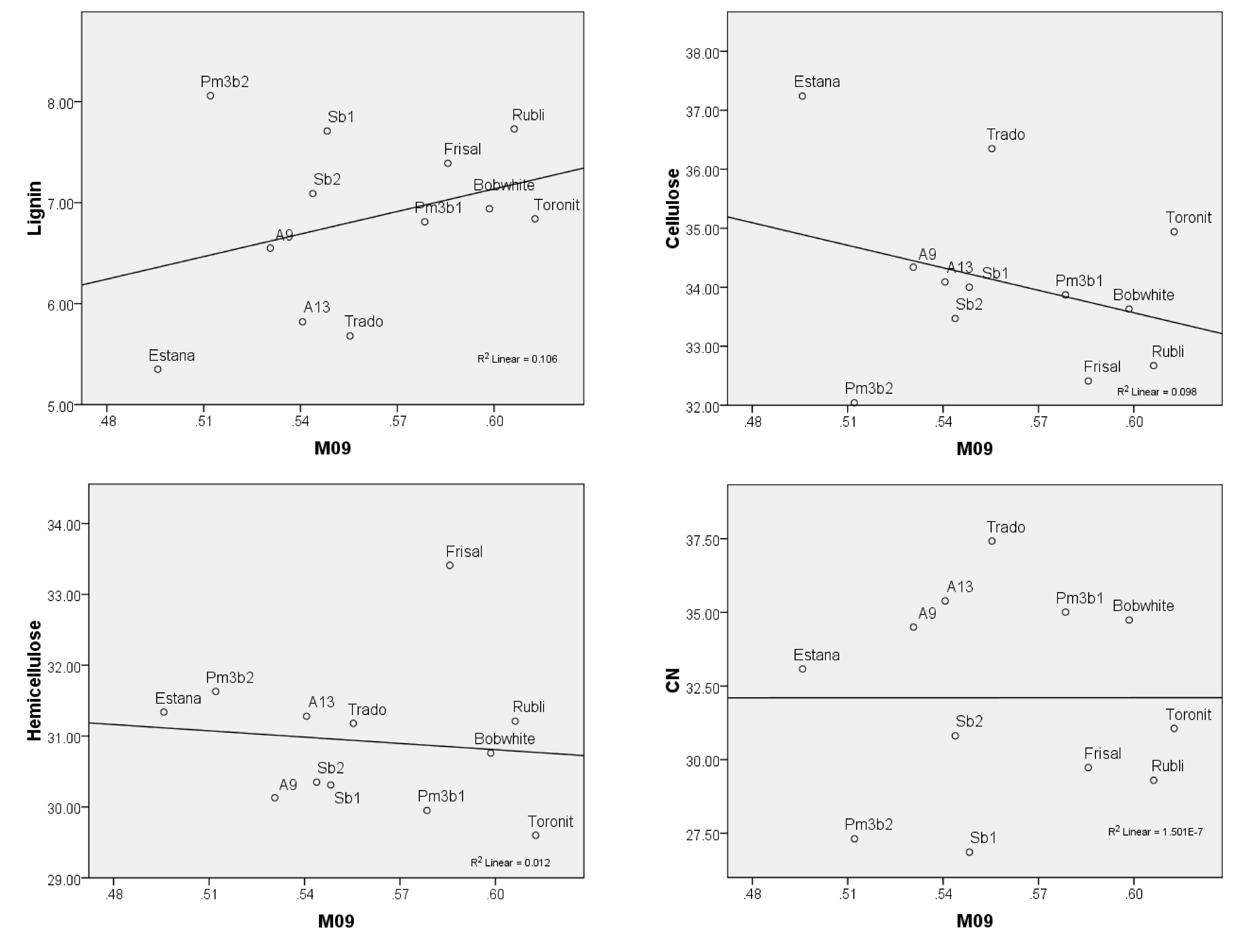

Supplement: Figure S2 — Correlation of decomposition rate M of the 2009 experiment and chemical parameters of all experimental varieties (N = 48 per variety). A Lignin B Cellulose C Hemicellulose and D C/N ratio. DW indicates dry weight. (TIFF) [file pone.0025014.s002.tiff]

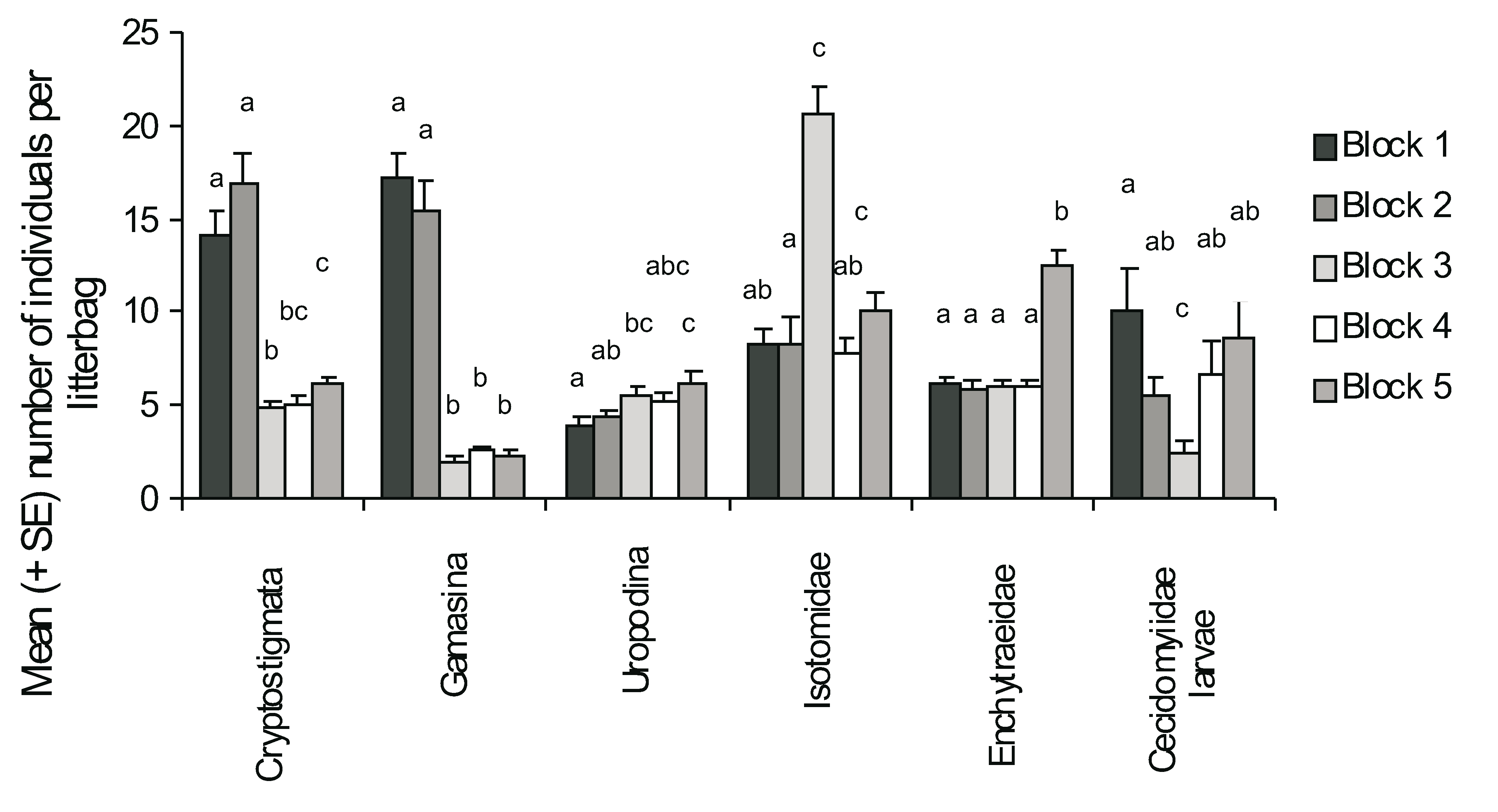

Supplement: Figure S3 — Block effect on soil fauna. Mean (+ SE) number of individuals per litterbag in the different blocks of the 2009 experiment (N = 144 per block). Letters above the columns indicate significant differences in taxa abundance among the blocks (Tukey HSD test, P<0.05). (TIFF) [file pone.0025014.s003.tiff]

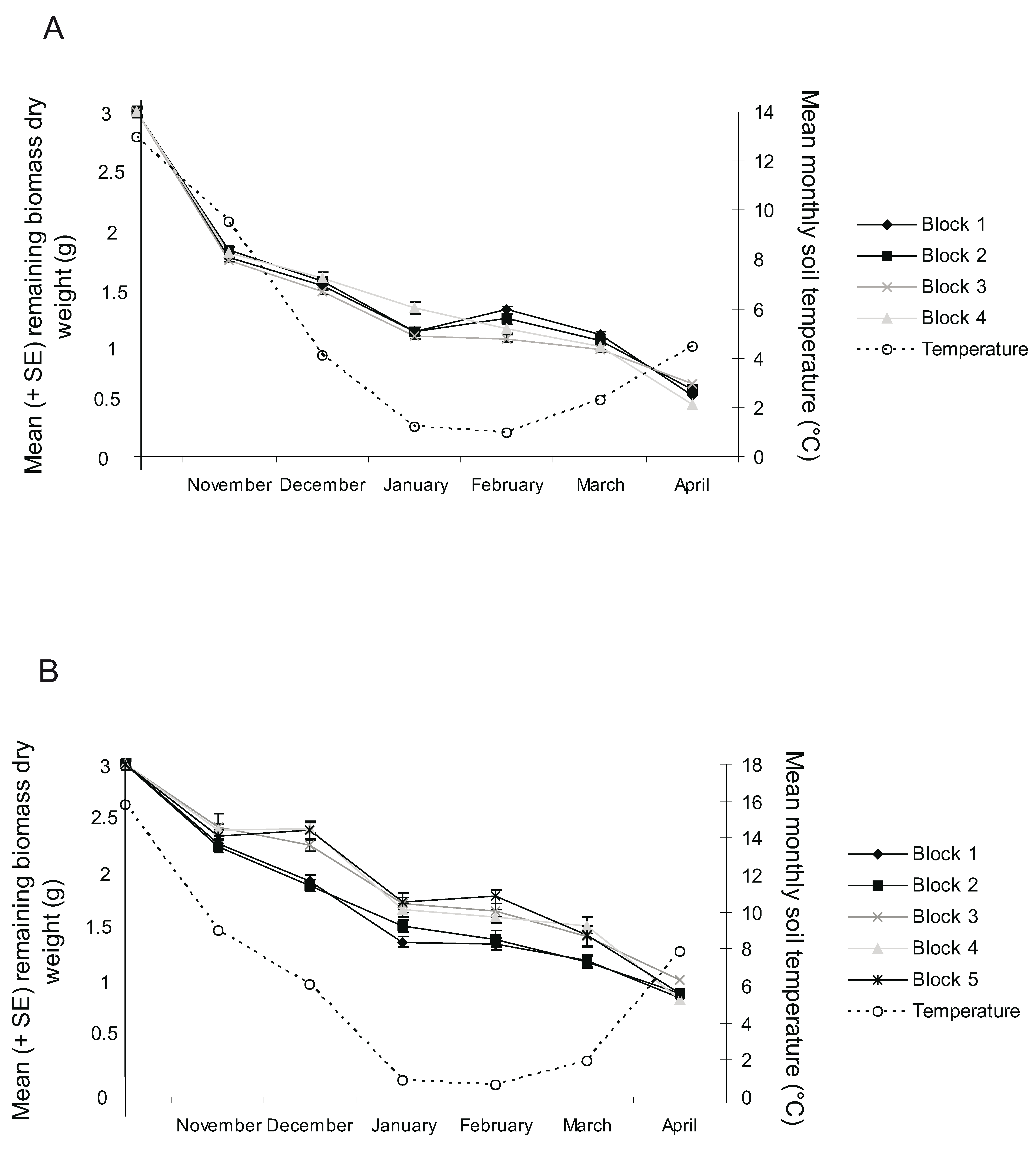

Supplement: Figure S4 — Block effect on decomposition. Mean (+ SE) remaining biomass dry weight in the blocks from November 2008 to April 2009 (A) (N = 24 per block and month) and from November 2009 to April 2010 (B) (N = 24 per block and month) and average monthly soil temperature curve at 5 cm depth. Asterisks show significant differences among the blocks (Tukey HSD test, P<0.05). Only significant differences are labelled. (TIFF) [file pone.0025014.s004.tiff]
