# Supplementary material for: No Adverse Effect of Genetically Modified Antifungal Wheat on Decomposition Dynamics and the Soil Fauna Community – A Field Study
Source: PLoS One. 2011 Oct 17;6(10):e25014. doi: 10.1371/journal.pone.0025014 (PMC3197184; doi:10.1371/journal.pone.0025014)
Supplement: Table S1 — Decomposition rate analysis. Results of the decomposition rate (M) analyses for the six months separately for the GM group (LME, with transgenic (Tr), resistance type (Rt) and their interaction as factors), the conventional cereal group and the wheat group (one-way ANOVA, with species or variety as factor). A 2008 experiment. B 2009 experiment. (DOC) [file pone.0025014.s005.doc]

**A**

*

*

*

|  |  | November | December | January | February | March | April |
| --- | --- | --- | --- | --- | --- | --- | --- |
| GM group | Transgenic (Tr) |  |  |  |  |  |  |
|  | *F*_1,2_ | 0.304 | 0.003 | 0.805 | 0.003 | 0.490 | 0.419 |
|  | *P* | 0.637 | 0.961 | 0.464 | 0.962 | 0.556 | 0.584 |
|  | Resistance type (Rt) |  |  |  |  |  |  |
|  | *F*_1,1_ | 4.692 | 2.187 | 0.129 | 0.109 | 0.093 | 0.382 |
|  | *P* | 0.275 | 0.379 | 0.780 | 0.797 | 0.812 | 0.647 |
|  | Tr x Rt |  |  |  |  |  |  |
|  | *F*_1,2_ | 1.196 | 0.965 | 0.002 | 0.001 | 0.910 | 0.345 |
|  | *P* | 0.388 | 0.492 | 0.968 | 0.977 | 0.441 | 0.616 |
| Conventional crop group | Species |  |  |  |  |  |  |
|  | *F*_2.37_ | 4.179 | 0.968 | 1.533 | 1.574 | 5.098 | 5.945 |
|  | *P* | 0.023 | 0.389 | 0.229 | 0.221 | 0.011 | 0.0061 |
|  |  |  |  |  |  |  |  |
| Wheat group | Variety |  |  |  |  |  |  |
|  | *F*_2,21_ | 3.231 | 1.024 | 2.087 | 1.419 | 0.019 | 2.969 |
|  | *P* | 0.060 | 0.377 | 0.149 | 0.264 | 0.981 | 0.073 |
|  |  |  |  |  |  |  |  |

**B**

|  |  | November | December | January | February | March | April |
| --- | --- | --- | --- | --- | --- | --- | --- |
| GM group | Transgenic (Tr) |  |  |  |  |  |  |
|  | *F*_1,2_ | 0.383 | 2.579 | 0.049 | 0.184 | 0.393 | 0.184 |
|  | *P* | 0.599 | 0.250 | 0.845 | 0.710 | 0.595 | 0.710 |
|  | Resistance type (Rt) |  |  |  |  |  |  |
|  | *F*_1,1_ | 0.320 | 0.255 | 0.736 | 0.003 | 2.218 | 0.003 |
|  | *P* | 0.673 | 0.703 | 0.549 | 0.966 | 0.376 | 0.966 |
|  | Tr x Rt |  |  |  |  |  |  |
|  | *F*_1,2_ | 2.569 | 0.346 | 0.096 | 0.433 | 1.910 | 0.433 |
|  | *P* | 0.250 | 0.616 | 0.786 | 0.578 | 0.301 | 0.578 |
| Conventional crop group | Species |  |  |  |  |  |  |
|  | *F*_2.47_ | 1.771 | 0.576 | 0.864 | 8.609 | 11.860 | 28.600 |
|  | *P* | 0.181 | 0.566 | 0.428 | <0.001 | <0.001 | <0.001 |
|  |  |  |  |  |  |  |  |
| Wheat group | Variety |  |  |  |  |  |  |
|  | *F*_2,27_ | 1.080 | 0.340 | 1.661 | 0.949 | 0.480 | 0.530 |
|  | *P* | 0.354 | 0.715 | 0.209 | 0.400 | 0.624 | 0.595 |
|  |  |  |  |  |  |  |  |
