# Supplementary material for: No Adverse Effect of Genetically Modified Antifungal Wheat on Decomposition Dynamics and the Soil Fauna Community – A Field Study
Source: PLoS One. 2011 Oct 17;6(10):e25014. doi: 10.1371/journal.pone.0025014 (PMC3197184; doi:10.1371/journal.pone.0025014)
Supplement: Table S2 — Mean (+ SE) remaining biomass dry weight of the different varieties. Results displayed from November 2008 to April 2009 (N = 8 per variety and month) and from November 2009 to April 2010 (N = 10 per variety and month). A GM group. B Conventional cereals. Different letters show significant differences among the varieties (Tukey HSD test, P<0.05). Only significant differences are labelled. (DOC) [file pone.0025014.s006.doc]

**A**

| Mean (± SE) remaining biomass dry weight (g) | | | | | | |
| --- | --- | --- | --- | --- | --- | --- |
| Variety | November 08 | December 08 | January 09 | February 09 | March 09 | April 09 |
|  |  |  |  |  |  |  |
| A9 | 2.03 ± 0.06 | 1.76 ± 0.09 | 1.08 ± 0.09 | 1.22 ± 0.14 | 0.91 ± 0.09 | 0.58 ± 0.08 |
| A13 | 1.93 ± 0.19 | 1.63 ± 0.21 | 1.26 ± 0.19 | 1.10 ± 0.14 | 0.93 ± 0.14 | 0.48 ± 0.13 |
| Frisal | 2.11 ± 0.13 | 1.61 ± 0.07 | 1.29 ± 0.15 | 1.16 ± 0.13 | 1.21 ± 0.14 | 0.71 ± 0.14 |
| Pm3b#1 | 1.86 ± 0.09 | 1.57 ± 0.10 | 1.18 ± 0.11 | 1.17 ± 0.10 | 1.13 ± 0.10 | 0.61 ± 0.14 |
| Sb#1 | 1.50 ± 0.08 | 1.58 ± 0.10 | 1.18 ± 0.21 | 1.05 ± 0.21 | 0.79 ± 0.16 | 0.48 ± 0.13 |
| Pm3#2 | 1.74 ± 0.06 | 1.33 ± 0.10 | 1.05 ± 0.09 | 1.08 ± 0.12 | 0.85 ± 0.06 | 0.42 ± 0.08 |
| Sb#2 | 1.82 ± 0.08 | 1.54 ± 0.09 | 1.30 ± 0.10 | 1.22 ± 0.13 | 1.17 ± 0.08 | 0.63 ± 0.10 |
| Variety | November 09 | December 09 | January 10 | February 10 | March 10 | April 10 |
|  |  |  |  |  |  |  |
| A9 | 2.20 ± 0.04 | 1.94 ± 0.23 | 1.62 ± 0.08 | 1.56 ± 0.14 | 1.43 ± 0.07 | 0.80 ± 0.09 |
| A13 | 2.37 ± 0.08 | 2.20 ± 0.12 | 1.65 ± 0.09 | 1.40 ± 0.06 | 1.23 ± 0.06 | 0.90 ± 0.10 |
| Frisal | 2.35 ± 0.05 | 2.36 ± 0.12 | 1.66 ± 0.08 | 1.63 ± 0.10 | 1.57 ± 0.08 | 0.97 ± 0.11 |
| Pm3b#1 | 2.41 ± 0.08 | 2.14 ± 0.14 | 1.63 ± 0.12 | 1.76 ± 0.12 | 1.40 ± 0.14 | 1.09 ± 0.11 |
| Sb#1 | 2.29 ± 0.04 | 2.19 ± 0.10 | 1.61 ± 0.08 | 1.52 ± 0.08 | 1.28 ± 0.11 | 0.99 ± 0.10 |
| Pm3#2 | 2.36 ± 0.07 | 1.98 ± 0.13 | 1.53 ± 0.11 | 1.37 ± 0.07 | 1.19 ± 0.10 | 0.79 ± 0.09 |
| Sb#2 | 2.27 ± 0.08 | 2.22 ± 0.11 | 1.49 ± 0.09 | 1.55 ± 0.11 | 1.26 ± 0.10 | 1.00 ± 0.15 |

**B**

| Mean (± SE) remaining biomass dry weight (g) | | | | | | |
| --- | --- | --- | --- | --- | --- | --- |
| Variety | November 08 | December 08 | January 09 | February 09 | March 09 | April 09 |
|  |  |  |  |  |  |  |
| Estana | 2.01 ± 0.04^a^ | 1.59 ± 0.04 | 1.15 ± 0.09 | 1.17 ± 0.16 | 0.74 ± 0.09^a^ | 0.34 ± 0.05^a^ |
| Trado | 2.02 ± 0.03^a^ | 1.72 ± 0.07 | 1.39 ± 0.10 | 1.26 ± 0.10 | 1.36 ± 0.18^b^ | 0.65 ± 0.10^b^ |
| Bobwhite | 1.98 ± 0.02^b^ | 1.70 ± 0.06 | 1.37 ± 0.10 | 1.51 ± 0.16 | 1.16 ± 0.11^b^ | 0.65 ± 0.07^b^ |
| Rubli | 1.71 ± 0.09^b^ | 1.51 ± 0.14 | 1.05 ± 0.09 | 1.55 ± 0.18 | 1.16 ± 0.19^b^ | 0.65 ± 0.12^b^ |
| Toronit | 1.61 ± 0.16^b^ | 1.54 ± 0.09 | 1.18 ± 0.13 | 1.23 ± 0.07 | 1.20 ± 0.13^b^ | 0.56 ± 0.08^b^ |
| Variety | November 09 | December 09 | January 10 | February 10 | March 10 | April 10 |
|  |  |  |  |  |  |  |
| Estana | 2.32 ± 0.04 | 2.16 ± 0.09 | 1.54 ± 0.10 | 1.33 ± 0.08^a^ | 1.09 ± 0.09^a^ | 0.48 ± 0.09^a^ |
| Trado | 2.35 ± 0.06 | 2.25 ± 0.06 | 1.66 ± 0.09 | 1.55 ± 0.04^ab^ | 1.42 ± 0.09^b^ | 0.76 ± 0.08^b^ |
| Bobwhite | 2.41 ± 0.06 | 2.31 ± 0.12 | 1.72 ± 0.07 | 1.68 ± 0.09^b^ | 1.51 ± 0.07^b^ | 1.13 ± 0.11^c^ |
| Rubli | 2.37 ± 0.04 | 2.31 ± 0.11 | 1.82 ± 0.06 | 1.74 ± 0.06^b^ | 1.54 ± 0.08^b^ | 1.13 ± 0.06^c^ |
| Toronit | 2.51 ± 0.09 | 2.20 ± 0.09 | 1.55 ± 0.16 | 1.90 ± 0.15^b^ | 1.63 ± 0.10^b^ | 1.24 ± 0.08^c^ |
