# Supplementary material for: No Adverse Effect of Genetically Modified Antifungal Wheat on Decomposition Dynamics and the Soil Fauna Community – A Field Study
Source: PLoS One. 2011 Oct 17;6(10):e25014. doi: 10.1371/journal.pone.0025014 (PMC3197184; doi:10.1371/journal.pone.0025014)
Supplement: Table S3 — Location analyses for the decomposition rate (M). Results displayed for the six months separately and for the complete data set of the 2008 experiment (one-way ANOVA with block as explanatory variable). (DOC) [file pone.0025014.s007.doc]

|  | |  | M |
| --- | --- | --- | --- |
| November | Block |  | |
|  | *F*_3,92_ | 2.071 | |
|  | *P* | 0.109 | |
| December | Block |  | |
|  | *F*_3,92_ | 2.983 | |
|  | *P* | 0.035 | |
| January | Block |  | |
|  | *F*_3,92_ | 5.670 | |
|  | *P* | 0.001 | |
| February | Block |  | |
|  | *F*_3,92_ | 1.829 | |
|  | *P* | 0.147 | |
| March | Block |  | |
|  | *F*_3,92_ | 0.501 | |
|  | *P* | 0.682 | |
| April | Block |  | |
|  | *F*_3,92_ | 0.788 | |
|  | *P* | 0.504 | |
| Overall | Block |  | |
|  | *F*_3,572_ | 1.513 | |
|  | *P* | 0.210 | |
