# Supplementary material for: No Adverse Effect of Genetically Modified Antifungal Wheat on Decomposition Dynamics and the Soil Fauna Community – A Field Study
Source: PLoS One. 2011 Oct 17;6(10):e25014. doi: 10.1371/journal.pone.0025014 (PMC3197184; doi:10.1371/journal.pone.0025014)
Supplement: Table S4 — Structural component analyses. Regression analysis between cellulose, C/N ratio, hemicellulose or lignin and decomposition rate) for the twelve varieties together. A 2008 experiment. B 2009 experiment. (DOC) [file pone.0025014.s008.doc]

**A**

|  | Cellulose | C/N ratio | Hemicellulose | Lignin |
| --- | --- | --- | --- | --- |
|  |  |  |  |  |
| *F*_1,574_ | 0.093 | 8.093 | 0.601 | 1.466 |
| *P* | 0.760 | 0.005 | 0.439 | 0.227 |
|  |  |  |  |  |

**B**

|  | Cellulose | C/N ratio | Hemicellulose | Lignin |
| --- | --- | --- | --- | --- |
|  |  |  |  |  |
| *F*_1,718_ | 2.520 | < 0.001 | 0.340 | 2.753 |
| *P* | 0.113 | 0.993 | 0.560 | 0.098 |
|  |  |  |  |  |
