# Supplementary material for: No Adverse Effect of Genetically Modified Antifungal Wheat on Decomposition Dynamics and the Soil Fauna Community – A Field Study
Source: PLoS One. 2011 Oct 17;6(10):e25014. doi: 10.1371/journal.pone.0025014 (PMC3197184; doi:10.1371/journal.pone.0025014)
Supplement: Table S5 — Mean (+ SE) remaining biomass dry weight of the different blocks. Results displayed from November 2008 to April 2009 (N = 8 per variety and month) and from November 2009 to April 2010 (N = 10 per variety and month). A 2008 experiment. B 2009 experiment. Different letters show significant differences among the varieties (Tukey HSD test, P<0.05). Only significant differences are labelled. (DOC) [file pone.0025014.s009.doc]

**A**

| Mean (± SE) Biomass dry weight (g) | | | | | | | |
| --- | --- | --- | --- | --- | --- | --- | --- |
| Location | November | December | January | February | March | April |  |
|  |  |  |  |  |  |  |  |
| Block 1 | 1.74 ± 0.04 | 1.48 ± 0.04^ab^ | 1.09 ± 0.03^a^ | 1.28 ± 0.04 | 1.06 ± 0.04 | 0.53 ± 0.03 |  |
| Block 2 | 1.80 ± 0.02 | 1.53 ± 0.02^ab^ | 1.08 ± 0.03^a^ | 1.21 ± 0.03 | 1.01 ± 0.03 | 0.58 ± 0.02 |  |
| Block 3 | 1.71 ± 0.03 | 1.44 ± 0.02^a^ | 1.05 ± 0.02^a^ | 1.03 ± 0.03 | 0.93 ± 0.04 | 0.63 ± 0.03 |  |
| Block 4 | 1.77 ± 0.05 | 1.55 ± 0.05^b^ | 1.29 ± 0.05^b^ | 1.11 ± 0.05 | 0.95 ± 0.04 | 0.46 ± 0.02 |  |

**B**

| Mean (± SE) Biomass dry weight (g) | | | | | | | |
| --- | --- | --- | --- | --- | --- | --- | --- |
| Location | November | December | January | February | March | April |  |
|  |  |  |  |  |  |  |  |
| Block 1 | 2.29 ± 0.04^ab^ | 1.95 ± 0.03^a^ | 1.40 ± 0.05^a^ | 1.37 ± 0.04^a^ | 1.22 ± 0.05^a^ | 0.89 ± 0.05 |  |
| Block 2 | 2.26 ± 0.04^a^ | 1.90 ± 0.06^a^ | 1.54 ± 0.06^ab^ | 1.42 ± 0.05^ab^ | 1.22 ± 0.07^a^ | 0.94 ± 0.06 |  |
| Block 3 | 2.44 ± 0.04^b^ | 2.27 ± 0.11^b^ | 1.74 ± 0.05^b^ | 1.68 ± 0.05^b^ | 1.45 ± 0.06^ab^ | 1.06 ± 0.09 |  |
| Block 4 | 2.40 ± 0.03^ab^ | 2.42 ± 0.05^b^ | 1.68 ± 0.05^ab^ | 1.62 ± 0.06^ab^ | 1.54 ± 0.06^b^ | 0.87 ± 0.07 |  |
| Block 5 | 2.36 ± 0.05^ab^ | 2.41 ± 0.05^b^ | 1.75 ± 0.08^b^ | 1.81 ± 0.09^b^ | 1.46 ± 0.06^ab^ | 0.93 ± 0.09 |  |
