# Supplementary material for: No Adverse Effect of Genetically Modified Antifungal Wheat on Decomposition Dynamics and the Soil Fauna Community – A Field Study
Source: PLoS One. 2011 Oct 17;6(10):e25014. doi: 10.1371/journal.pone.0025014 (PMC3197184; doi:10.1371/journal.pone.0025014)
Supplement: Table S6 — Soil parameters analyses. Done for the different blocks. A 2008 experiment. B 2009 experiment. (DOC) [file pone.0025014.s010.doc]

**A**

| Soil parameters | Block 1 | Block 2 | Block 3 | Block 4 |
| --- | --- | --- | --- | --- |
| pH (H_2_0) | 7.2 | 7.1 | 7.0 | 7.2 |
| Humus (%) | 3.8 | 3.6 | 3.6 | 3.6 |
| Clay (%) | 32.4 | 31.4 | 31.8 | 31.4 |
| Silt (%) | 29.5 | 30.6 | 30.3 | 28.7 |
| Sand (%) | 34.3 | 34.4 | 34.3 | 36.0 |

**B**

| Soil parameters | Block 1 | Block 2 | Block 3 | Block 4 | Block 5 |
| --- | --- | --- | --- | --- | --- |
| pH (H_2_0) | 6.9 | 7.5 | 6.6 | 6.5 | 6.5 |
| Humus (%) | 3.2 | 3.5 | 3.4 | 3.4 | 4.2 |
| Clay (%) | 26.7 | 24.3 | 28.9 | 28.7 | 32.8 |
| Silt (%) | 32.9 | 24.9 | 32.3 | 33.7 | 33.2 |
| Sand (%) | 37.2 | 47.3 | 35.4 | 34.2 | 29.8 |
